# Low Phytanic Acid-Concentrated DHA Prevents Cognitive Deficit and Regulates Alzheimer Disease Mediators in an ApoE^−/−^ Mice Experimental Model

**DOI:** 10.3390/nu11010011

**Published:** 2018-12-20

**Authors:** María Belén Ruiz-Roso, Víctor Echeverry-Alzate, Baltasar Ruiz-Roso, José Carlos Quintela, Sandra Ballesteros, Vicente Lahera, Natalia de las Heras, José Antonio López-Moreno, Beatriz Martín-Fernández

**Affiliations:** 1 Department of Physiology, Faculty of Medicine, Complutense University, 28040 Madrid, Spain; mbruizroso@ucm.es (M.B.R.-R.); sballest@ucm.es (S.B.); vlahera@med.ucm.es (V.L.); nataliaheras@med.ucm.es (N.d.l.H.); 2 Department of Psychobiology, Faculty of Psychology, Complutense University, 28223 Madrid, Spain; echeverry.v@psi.ucm.es; 3 Department of Nutrition and Food Science, Faculty of Pharmacy, Complutense University, 28040 Madrid, Spain; ruizrojo@ucm.es; 4 Natac Pharma S.L., Alcorcón, 28923 Madrid, Spain; jcquintela@natacgroup.com; 5 Natac Biotech S.L., Alcorcón, 28923 Madrid, Spain

**Keywords:** Alzheimer’s, DHA, ApoE^−/−^, phytanic acid, inflammation, neuroprotection, oxidation

## Abstract

Alzheimer’s disease (AD) is the main cause of dementia and cognitive impairment. It has been associated with a significant diminution of omega-3 polyunsaturated fatty acid docosahexaenoic acid (DHA) levels in the brain. Clinical trials with DHA as a treatment in neurological diseases have shown inconsistent results. Previously, we reported that the presence of phytanic acid (PhA) in standard DHA compositions could be blunting DHA’s beneficial effects. Therefore, we aimed to analyze the effects of a low PhA-concentrated DHA and a standard PhA-concentrated DHA in Apolipoprotein E knockout (ApoE^−/−^) mice. Behavioral tests and protein expression of pro-inflammatory, pro-oxidant, antioxidant factors, and AD-related mediators were evaluated. Low PhA-concentrated DHA decreased Aβ, ß-amyloid precursor protein (APP), p-tau, Ca^2+^/calmodulin-dependent protein kinase II (CAMKII), caspase 3, and catalase, and increased brain derived neurotrophic factor (BDNF) when compared to standard PhA-concentrated DHA. Low PhA-concentrated DHA decreased interleukin (IL)-6 and tumor necrosis factor alpha (TNF-α) protein expression in ApoE^−/−^ mice when compared to standard PhA-concentrated DHA. No significant differences were found in p22phox, inducible nitric oxide synthase (iNOS), glutathione peroxidase (GPx), superoxide dismutase 1 (SOD-1), and tau protein expression. The positive actions of a low PhA-concentrated DHA were functionally reflected by improving the cognitive deficit in the AD experimental model. Therefore, reduction of PhA content in DHA compositions could highlight a novel pathway for the neurodegeneration processes related to AD.

## 1. Introduction

Alzheimer’s disease (AD) is the main cause of dementia and one of the greatest healthcare challenges of the 21st century [1]. The clinical symptoms of AD might be caused by an extensive loss of synapses and neurons leading to a strong hippocampal and cortical atrophy [2,3] reflected in processes of memory formation and storage [4,5]. The past 30 years of AD research have produced substantial evidence that accumulation of abnormally folded Aβ and tau proteins in amyloid plaques and neuronal tangles are causally related to neurodegenerative processes in patients’ brains [6]. Aβ peptides are an aggregation-prone secreted peptide generated by sequential proteolytic processing of the ß-amyloid precursor protein (APP) [7]. The amyloid precursor protein is a ubiquitously expressed type I-transmembrane protein cycling between the plasma membrane and acidic intracellular compartments [8,9,10]. Secondary to the buildup of plaques of Aβ, AD is characterized by neurofibrillary tangles of hyperphosphorylated tau [11]. There is also significant evidence that intracellular calcium (Ca^2+^) homeostasis is disrupted in AD and can exacerbate Aβ formation and promote tau hyperphosphorylation [12]. The Ca^2+^/calmodulin (CaM)-dependent protein kinase II (CaMKII) is the major post-synaptic protein at excitatory synapses and fundamentally important for synaptic plasticity and memory formation [13,14]. Inflammatory processes and increased oxidative stress have also been proposed to highly contribute to Aβ neurotoxicity [7]. Neuroinflammation from aberrantly activated glia is reemerging as an important mechanism that contributes to AD progression involving TNF-α, cyclooxygenase-2 (COX-2), and interleukin 6 (IL-6) amongst others [15,16]. In this regard, intake of antioxidants has been proposed to reduce AD risk by decreasing the risk of cerebrovascular disease [7,17].

In recent years, essential omega-3 (ω-3) long-chain polyunsaturated fatty acid (LCPUFA) has been used in AD treatment because of their antioxidant properties [18,19]. Docosahexaenoic acid (DHA, 22:6 *n*-3), the most predominant LCPUFA found at the second position in phospholipids on neuronal and synaptic membranes [20], reduces oxidative stress and possess favorable effects on neuronal and vascular functions and inflammatory processes [21,22]. Besides, DHA participates in normal brain growth, development, and function [23], acting as a neurotrophic factor [24] and modulating synaptic activity [25]. There is a growing body of evidence that shows the relationship between DHA and memory. Lower DHA has been associated with cognitive impairment in such a way that plasma phosphatidylcholine DHA is a significant predictor of memory functioning [26]. Other studies have revealed that DHA treatment can improve memory in animal models of AD [27,28,29] and in healthy humans of all ages [30,31], and in adults with age-related cognitive impairments [32,33]. However, there are still some inconsistencies in findings from clinical and pre-clinical studies with DHA as a therapy in neurological diseases [34]. In a previous study, we proposed the presence of phytanic acid (PhA) in standard DHA treatments as a cause of DHA positive effects lack [35]. Standard DHA supplements contain high concentrations of the branched-chain phytanic acid (3,7,11,15-tetramethylhexadecanoic acid, PhA) which has been found to disturb the integrity of neural cells [36]. Phytanic acid is naturally found in oily fish and has important cytotoxic and pro-oxidant activity and induces apoptosis in neurons, photoreceptors, astrocytes, cochlea, Purkinje cells, vascular endothelium, and hepatocytes [36]. However, even though the presence of PhA in standard DHA supplements is common, currently there are no studies which have investigated how the effects of DHA on cognitive function are regulated by the concentration of PhA. In our study, carried out in activated microglial cells, we proposed that PhA in DHA treatments might be undermining the benefits of DHA by decreasing cell viability and inducing oxidative stress, inflammation, and decreased neuroprotective mediators expression [35]. 

Apolipoprotein E (ApoE) is a regulator of cholesterol metabolism secreted in the central nervous system (CNS) by astrocytes [37,38], and it is the major lipoprotein transporter in the CNS [39,40]. Cholesterol was identified as an early risk factor for AD [41], indicating an important role approximately at the same time when amyloid deposition initiates. Moreover, it was early proposed that ApoE^−/−^ mice have highly increased plasma lipid levels [42,43], which may independently cause synaptic dysfunction and cognitive deficits [44]. Therefore, Apo E^−/−^ mice have been validated as an experimental AD model [45]. 

In view of the aforementioned, we aimed to analyze and compare the effects of a low phytanic acid-concentrated DHA with a standard phytanic acid-concentrated DHA in ApoE^−/−^ mice fed on a high-fat diet. Effects and comparison of the treatments on behavioral tests (locomotor activity and spatial learning and memory) and protein expression of pro-inflammatory, pro-oxidant, antioxidant factors, Aβ, APP, tau, p-tau, and brain derived neurotrophic factor (BDNF) were explored.

## 2. Materials and Methods

### 2.1. Ethical Approval

The study was conducted in accordance with the Declaration of Helsinki, and the protocol was approved by the Ethics Committee of Universidad Complutense and granted and approved by the Universidad Complutense Ethics Review Board following the National Guideline 53/2013 (Project identification code RD20160028).

### 2.2. Experimental Design and Animal Model

The study was conducted in 54 male mice (Taconic Biosciences Inc., Bomholtvej, Denmark). ApoE^−/−^ mice were fed a high-fat diet (*n* = 9 per group): (1) ApoE^−/−^, (2) ApoE^−/−^ + DHA 50 ppm of PhA (ApoE+DHA (PhA:50)), and (3) ApoE^−/−^ + DHA 1000 ppm of PhA (ApoE+DHA (PhA:1000)). Wild-type C57BL/6 mice were fed a normal chow diet: (4) Control group (Control), (5) DHA 50 ppm of PhA (DHA (PhA:50)), and (6) DHA (PhA:1000). Both compositions of DHA–PhA were added to the fat diet (refined olive oil) at 10% (Natac Pharma, S.L. Madrid, Spain). The treatment period was 10 weeks. Mice were kept in a quiet room at constant temperature (20–22 °C) and humidity (50–60%). Full diet composition is provided in Supplementary Materials Table S1.

### 2.3. Behavioral Studies

Behavioral experiments were performed during the dark cycle (lights off at 9:00), with dimly light for video recording.

#### 2.3.1. Locomotor Activity

Spontaneous locomotor activity was evaluated using custom-made boxes (35 × 35 × 30 cm) equipped with 8 photocells arranged in 2 lines (1 and 5 cm above the floor), and the locomotor activity (horizontal and vertical) was detected as beam breaks. Twenty-four hours before testing, all animals remained 30 min (individually housed) in the test room followed by 15 min in the apparatus to facilitate context habituation. On the test day, animals were placed in the test room for at least 30 min before testing. Then, the locomotor activity was registered during a single 30-min trial [46,47].

#### 2.3.2. Morris Water Maze

Spatial learning and memory were assessed using the Morris Water Maze (MWM) as previously described in detail [48,49]. Basically, the maze was a circular pool (diameter 122 cm, height 40 cm) filled with 23 ± 1 °C water, located in a room with visible external cues, and monitored by a video camera above the apparatus. A hidden escape platform (diameter 10 cm and height 12 cm) was submerged 1 cm below the water surface in one of the four equal imaginary quadrants. From day 1 to 5 (learning curve), the animals were trained to find the escape platform, with 4 trials per day, a time limit of 60 s per trial, and a 4–5 min interval between trials (their escape latencies were recorded for each trial). To assess reference memory (probe trial), 24 h after the last learning day, one trial without platform was carried out for 60 s with a novel start position in the maze to ensure that the mice remembered the goal location rather than a specific swim path. An experimenter blind to the treatment scored the latency time to reach the target site (the previous platform location) and the time spent within a 10 cm target annulus around the former platform location [50]. 

After the spatial version of the MWM (days 7 to 9), the animals were tested for their motivation to escape from the water, no spatial learning, and sensorimotor abilities in a cued learning. Mice were trained to find the submerged platform indicated by a visible local cue. All animals received 4 trials over three consecutive days in which the cued platform and the start position were moved to a new location on every trial. 

### 2.4. Serum Analysis

Serum total cholesterol and low-density lipoprotein cholesterol (LDL-c) levels were measured by Spectrophotometric techniques (Vitros Fusion 5.1, Diagnostics OrthoClinical, Johnson & Johnson, New York, NY, USA). 

### 2.5. Western Blot Analysis

#### 2.5.1. Isolation of Total Proteins

Frozen hippocampus samples (100 mg) were homogenized using Allprep^®^ DNA/RNA/Protein Mini Kit (Quiagen, Hilden, Germany). The extracts were aliquoted and stored at −80 °C for Western blotting analysis.

#### 2.5.2. Western Blot Analysis

Proteins were separated on SDS-PAGE gels under reducing conditions and transferred to nitrocellulose membranes (Bio-Rad, Hercules, CA, USA). The membranes were blocked for 1 h with 5% (*w*/*v*) bovine serum albumin (BSA) as a blocking agent (Sigma, Madrid, Spain) in phosphate buffered saline (PBS) solution with the detergent Tween 20 (PBST; 1% PBS, 0.1%Tween 20 *v*/*v*) at room temperature. After washing with PBST, the membranes were probed overnight at 4 °C with appropriate primary antibodies: ß-amyloid precursor protein (APP) (Abcam, Cambridge, UK), ß-amyloid peptide (Aß) (Abcam, Cambridge, UK), interleukin-6 (IL-6) (Abcam, Cambridge, UK), tumor necrosis factor alpha (TNF-α, 1:1000) (Abcam, Cambridge, UK), nicotinamide adenine dinucleotide phosphate p22phox subunit (p22NADPH, 1:500) (Santa Cruz Biotechnology, Heidelberg, Germany), superoxide dismutase 1 (SOD-1, 1:2500) (Abcam, Cambridge, UK), catalase (Cat, 1:750) (Abcam, Cambridge, UK), caspase-3 (1:1000) (Abcam, Cambridge, UK), brain derived neurotrophic factor (BDNF, 1:200) (Santa Cruz Biotechnology, Heidelberg, Germany), glutathione peroxidase (GPx, 1:1000) (Abcam, Cambridge, UK), tau protein (Total tau, 1:5000) (Abcam, Cambridge, UK), phospho tau protein-Ser 396 (Tau ^S-396^, 1:250) (Abcam, Cambridge, UK), and inducible nitric oxide synthase (iNOS, 1:300) (Abcam, Cambridge, UK). After washing, the membrane was incubated for 1 h with peroxidase-conjugated rabbit or mouse anti-goat IgG secondary antibody (1:10,000). For detection, an ECL Advance Western Blotting Detection kit (Amersham Bioscience, Amersham, UK) was used. Blots were probed with rabbit monoclonal anti-GAPDH antibody (1:10,000, Abcam, Cambridge, UK) or rabbit monoclonal anti-Beta actin antibody (1:10,000, Abcam, Cambridge, UK) as internal control, to normalize between gels. Quantification was expressed as a percentage of relative protein expression (protein/GAPDH or Beta-actin) vs. control group.

### 2.6. Statistical Analysis

**Figure 1:**
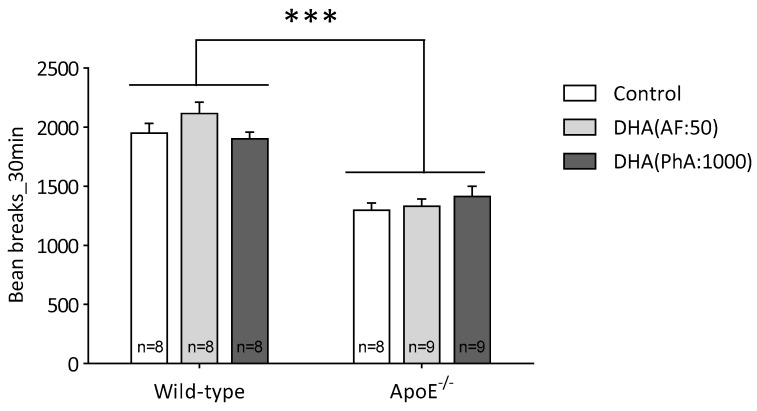
Effects of docosahexaenoic acid (DHA)(PhA:50) and DHA (PhA:1000) on spontaneous mouse locomotor activity in ApoE^−/−^ and wild-type mice. Data shown as mean ± SEM. *** *p* < 0.0001 compared between genotypes. (*n* = 8 or 9).

**Figure 2:**
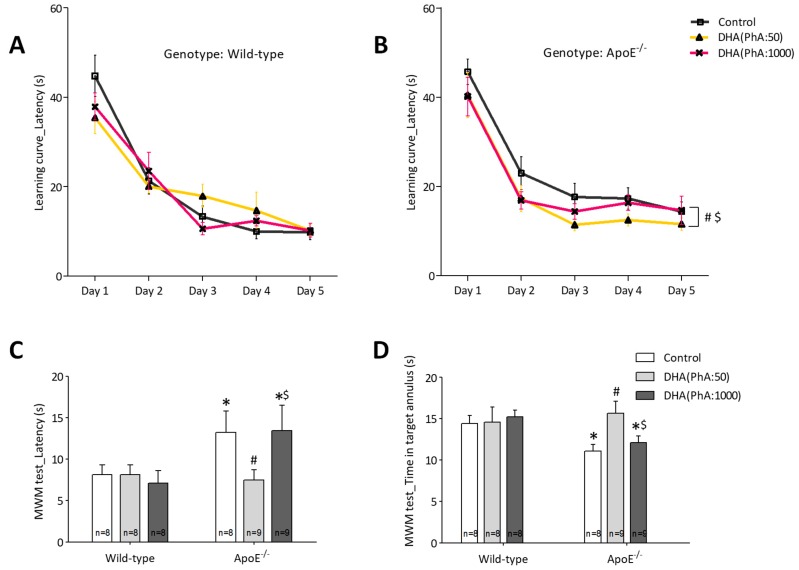

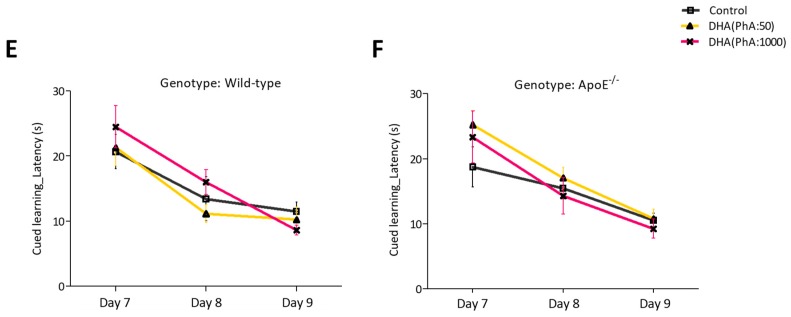
Effects of DHA (PhA:50) and DHA (PhA:1000) on the curve learning (**A**,**B**), spatial memory (**C**,**D**), and visual cue localization (**E**,**F**) in the Morris Water Maze (MWM) test in ApoE^−/−^ and wild-type mice. Data shown as mean ± SEM. * *p* < 0.05 compared to control wild-type mice; ^#^ *p* < 0.05 compared to control ApoE^−/−^ mice; ^$^ *p* < 0.05 compared between DHA (PhA:50) and DHA (PhA:1000) in ApoE^−/−^ mice. (*n* = 8 or 9).

**Figure 3:**
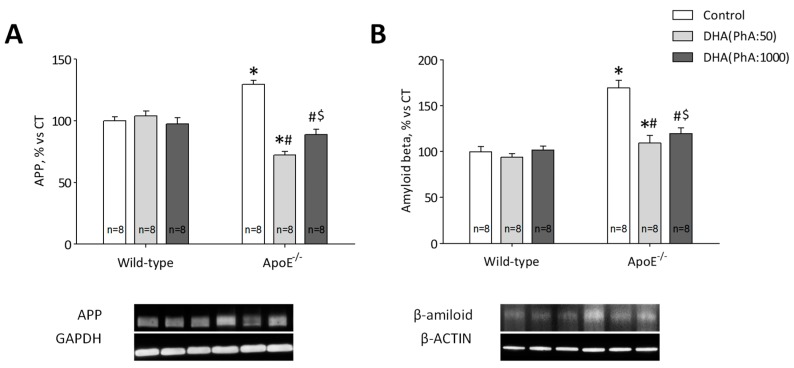
Effects of DHA (PhA:50) and DHA (PhA:1000) on relative protein expression of (**A**) amyloid precursor protein (APP) and (**B**) amyloid beta (Aß) in hippocampus of ApoE^−/−^ and wild-type mice. Data show mean ± SEM. * *p* < 0.05 compared to control wild-type mice; ^#^ *p* < 0.05 compared to control ApoE^−/−^ mice; ^$^ *p* < 0.05 compared between DHA (PhA:50) and DHA (PhA:1000) in ApoE^−/−^ mice. (*n* = 8).

**Figure 4:**
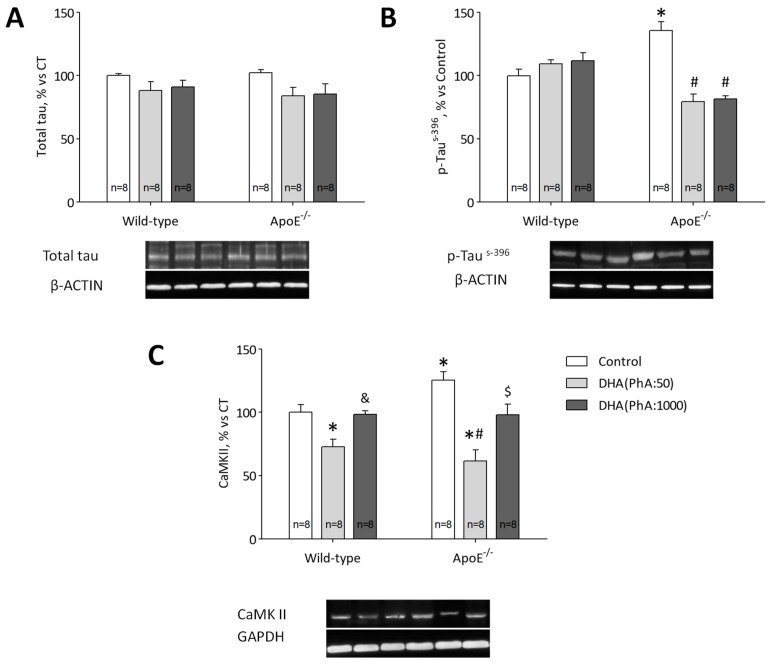
Effects of DHA (PhA:50) and DHA (PhA:1000) on relative protein expression of (**A**) Total tau, (**B**) p-Tau^s-396^ and (**C**) Ca^2+^/calmodulin-dependent protein kinase II (CaMK II) in hippocampus of ApoE^−/−^ and wild-type mice. Data show mean ± SEM. * *p* < 0.05 compared to control wild-type mice; ^#^ *p* < 0.05 compared to control ApoE^−/−^ mice; ^&^ *p* < 0.05 compared between DHA (PhA:50) and DHA (PhA:1000) in wild-type mice; ^$^ *p* < 0.05 compared between DHA (PhA:50) and DHA (PhA:1000) in ApoE^−/−^ mice. (*n* = 8).

**Figure 5:**
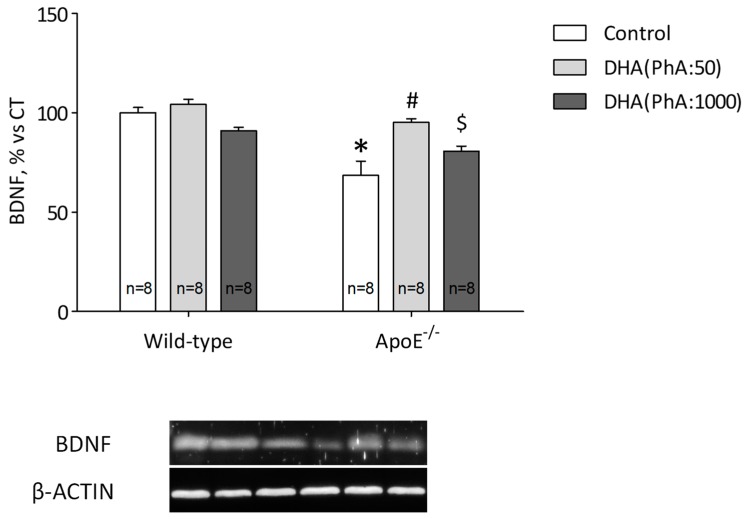
Effects of DHA (PhA:50) and DHA (PhA:1000) on relative protein expression brain derived neurotrophic factor (BDNF) in hippocampus of ApoE^−/−^ and wild-type mice. Data show mean ± SEM. * *p* < 0.05 compared to control wild-type mice; ^#^ *p* < 0.05 compared to control ApoE^−/−^ mice; ^$^ *p* < 0.05 compared between DHA (PhA:50) and DHA (PhA:1000) in ApoE^−/−^ mice. (*n* = 8).

**Figure 6:**
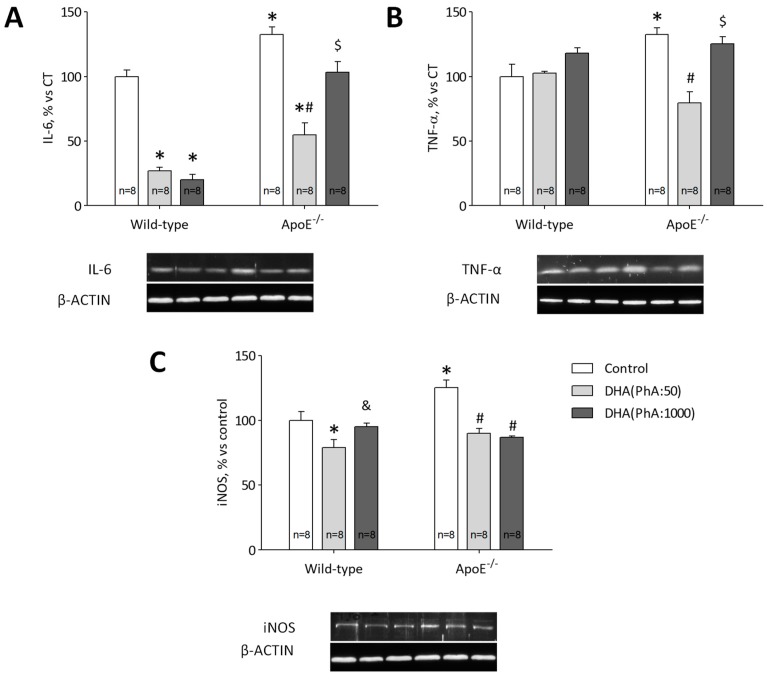
Effects of DHA (PhA:50) and DHA (PhA:1000) on relative protein expression of (**A**) interleukin-6 (IL-6), (**B**) tumor necrosis factor alpha (TNF-α) and (**C**) inducible nitric oxide synthase (iNOS) in hippocampus of ApoE^−/−^ and wild-type mice. Data shown as mean ± SEM. * *p* < 0.05 compared to control wild-type mice; ^#^ *p* < 0.05 compared to control ApoE^−/−^ mice; ^&^ *p* < 0.05 compared between DHA (PhA:50) and DHA (PhA:1000) in wild-type mice; ^$^ *p* < 0.05 compared between DHA (PhA:50) and DHA (PhA:1000) in ApoE^−/−^ mice. (*n* = 8).

**Figure 7:**
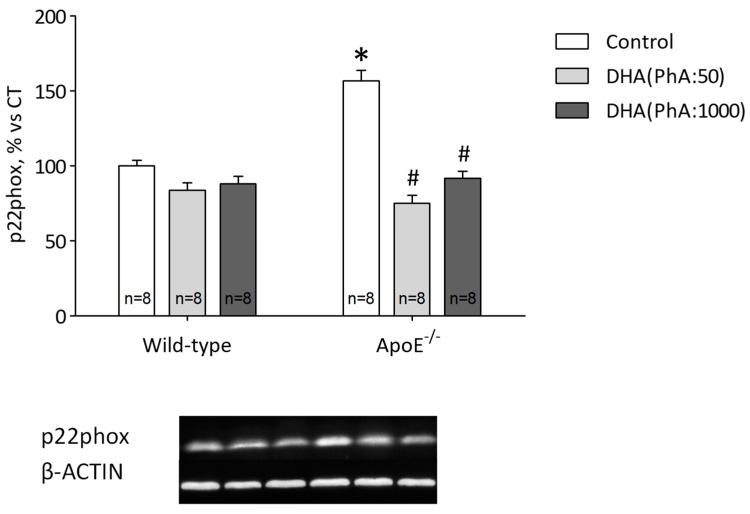
Effects of DHA (PhA:50) and DHA (PhA:1000) on relative protein expression of NADPH oxidase subunit p22phox (p22phox) in hippocampus of ApoE^−/−^ and wild-type mice. Data shown as mean ± SEM. * *p* < 0.05 compared to control wild-type mice; ^#^ *p* < 0.05 compared to control ApoE^−/−^ mice. (*n* = 8).

**Figure 8:**
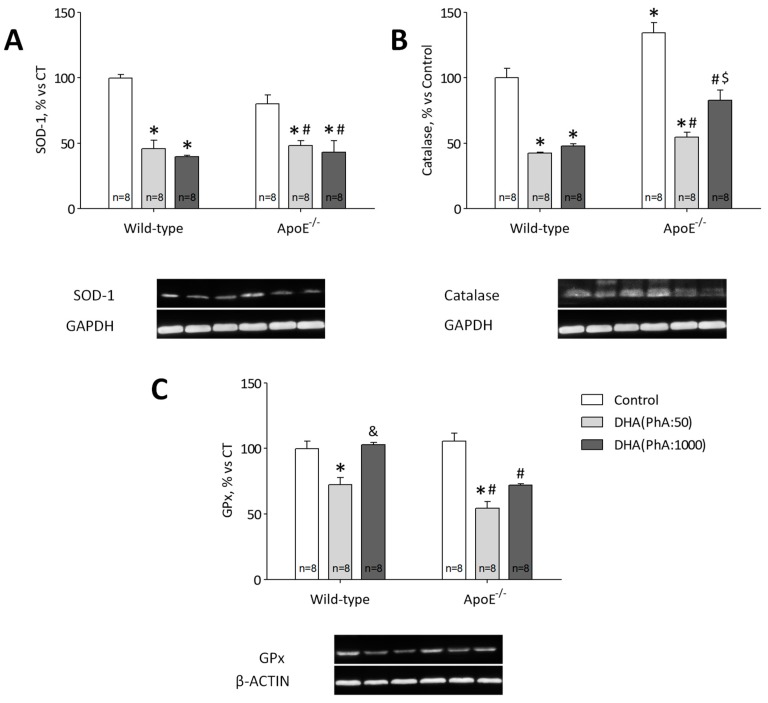
Effects of DHA (PhA:50) and DHA (PhA:1000) on relative protein expression of (**A**) superoxide dismutase 1 (SOD-1), (**B**) catalase, and (**C**) glutathione peroxidase (GPx) in hippocampus of ApoE^−/−^ and wild-type mice. Data show mean ± SEM. * *p* < 0.05 compared to control wild-type mice; ^#^ *p* < 0.05 compared to control ApoE^−/−^ mice; ^&^ *p* < 0.05 compared between DHA (PhA:50) and DHA (PhA:1000) in wild-type mice; ^$^ *p*<0.05 compared between DHA (PhA:50) and DHA (PhA:1000) in ApoE^−/−^ mice. (*n* = 8).

**Figure 9:**
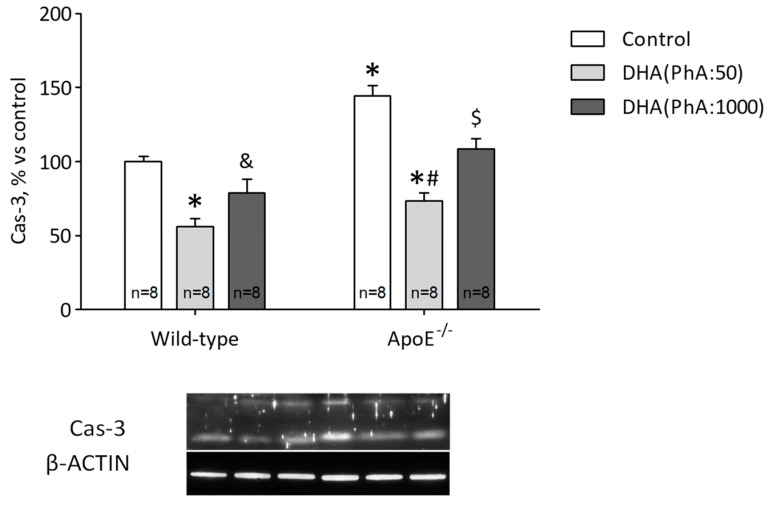
Effects of DHA (PhA:50) and DHA (PhA:1000) on relative protein expression of caspase-3 (cas-3) in hippocampus of ApoE^−/−^ and wild-type mice. Data shown as mean ± SEM. * *p* < 0.05 compared to control wild-type mice; ^#^ *p* < 0.05 compared to control ApoE^−/−^ mice; ^&^ *p* < 0.05 compared between DHA (PhA:50) and DHA (PhA:1000) in wild-type mice; ^$^ *p* < 0.05 compared between DHA (PhA:50) and DHA (PhA:1000) in ApoE^−/−^ mice. (*n* = 8).

Data were presented as mean ± standard error of the mean (SEM). Data from Figure 1, Figure 2C,D, Figure 3, Figure 4, Figure 5, Figure 6, Figure 7, Figure 8 and Figure 9, were analyzed using a two-way analysis of variance (ANOVA) (genotype × DHA). The results were followed by Bonferroni’s test. Data from Figure 2A,B,E,F were analyzed using a two-way mixed ANOVA (within-mice: trials, between-groups: diet). A significance level of *p* < 0.05 was applied to all ANOVA statistical analyses, and when significant, the results were followed by Tukey’s post-hoc tests. The GraphPad Prism 6 (version 6.07; Graph Pad Software Inc. San Diego, CA, USA) was used for all statistical analyses.

## 3. Results

### 3.1. DHA (PhA:50) Improved Spatial Memory in ApoE^−/−^ Mice Compared to DHA (PhA:1000)

#### 3.1.1. Locomotor Activity

As shown in Figure 1, ApoE^−/−^ caused a significant reduction on the spontaneous locomotor activity of the animals (two-way ANOVA: genotype *F*(1,44) = 108.99, *p* < 0.0001; DHA *F*(2,44) = 0.89, not significant (NS); interaction *F*(2,44) = 1.97, NS). During the 30-min period observed, the mean of the activity of the ApoE groups was 32.1% lower than the control groups. No PhA concentration could reverse these effects.

#### 3.1.2. Learning and Memory

Figure 2A,B depicts the curve learning in the MWM test. Whereas the concentration of DHA had no significant effects on the control groups, the DHA (PhA:50) supplementation was able to revert the deficits in the learning curve caused by the ApoE mutation (two-way ANOVA: days *F*(4,92) = 54.18, *p* < 0.0001; DHA *F*(2,23) = 3.87, *p* < 0.05; NS; interaction *F*(8,92) = 0.29, NS). On the test day, Figure 2C, it was shown that some of the control groups differed on their latency to reach the hidden escape platform. However, the ApoE mutation caused memory to worsen over time as it was revealed by a greater latency to find the hidden platform. This detrimental effect on the animal’s memory was prevented by the DHA (PhA:50) treatment. The same effect is shown in Figure 2D. It reveals the time that the animals spent on the annulus close to the escape platform. Spending more time on it indicates that the animals closely remembered the zone where the hidden escape platform was. Docosahexaenoic acid (PhA:50) treatment increased the time on the annulus, matching the time that the control animals passed on the annulus (two-way ANOVA: genotype *F*(1,44) = 8.13, *p* < 0.01; DHA *F*(2,44) = 0.83, NS; interaction *F*(2,44) = 7.84, *p* < 0.005). After the test day, it was examined whether the animals could find the platform marked with a clear visual cue, therefore, no spatial memory was needed. There were not significant differences in any of the treated groups, Figure 2E,F (i.e., two-way ANOVA: genotype *F*(2,46) = 0.63, NS; DHA *F*(2,23) = 1.36, NS; interaction *F*(4,46) = 0.62, NS).

### 3.2. Effects of DHA (PhA:50) on Body Weight and Lipid Profile of ApoE^−/−^ Mice Compared to DHA (PhA:1000)

Serum concentrations of total cholesterol and LDL-c were significantly increased in ApoE^−/−^ mice compared to the control group and decreased in ApoE^−/−^ + DHA (PhA:50) and ApoE^−/−^ + DHA (PhA:1000) mice compared to those of the ApoE^−/−^ group. Similarly, serum concentrations of total cholesterol and LDL-c were significantly decreased in DHA (PhA:50) and DHA (PhA:1000) mice compared to the control group (Table 1). Table 1 shows total cholesterol (two-way ANOVA: genotype *F*(1,37) = 970.96, *p* < 0.0001; DHA *F*(2,37) = 11.92, *p* < 0.0001; interaction *F*(2,37) = 1.05, NS). Table 2 shows LDL-c (two-way ANOVA: genotype *F*(1,39) = 995.37, *p* < 0.0001; DHA *F*(2,39) = 7.68, *p* < 0.005; interaction *F*(2,39) = 3.80, *p* < 0.05).

No significant differences were found in body weight in studied experimental groups (Table 2).

### 3.3. DHA (PhA:50) Decreased Hippocampal Protein Expression of APP and Aß Compared to DHA (PhA:1000) in ApoE^−/−^ Mice

ApoE^−/−^ mice showed increased hippocampal APP protein expression compared to the control (*p* < 0.001). DHA (PhA:50) normalized (*p* < 0.001) APP hippocampal protein expression in ApoE^−/−^ mice whereas ApoE^−/−^ + DHA (PhA:1000) group showed increased (*p* < 0.05) levels of APP compared to ApoE^−/−^ + DHA (PhA:50) group. No significant differences were found in APP protein expression in DHA (PhA:50) and DHA (PhA:1000) groups versus Control group (Figure 3A). Figure 3A (two-way ANOVA: genotype *F*(1,42) = 1.36, *p* < 0.001; DHA *F*(2,42) = 28.16, *p* < 0.001; interaction *F*(4,42) = 33.65, *p* < 0.001).

Protein expression of Aß was increased in hippocampus of ApoE^−/−^ group compared to Control (*p* < 0.001). Both DHA treatment, DHA (PhA:50) and DHA (PhA:1000), decreased expression of Aß in hippocampus of ApoE^−/−^ mice versus ApoE^−/−^ group (*p* < 0.001). Although, the hippocampal Aß level was significantly higher in ApoE^−/−^ + DHA (PhA:1000) group than in the ApoE^−/−^ + DHA (PhA:50) group (*p* < 0.001). No significant differences were found in Aß expression in DHA (PhA:50) and DHA (PhA:1000) groups versus Control group (Figure 3B). Figure 3B (two-way ANOVA: genotype *F*(1,37) = 41.64, *p* < 0.001; DHA *F*(2,37) = 14.99, *p* < 0.001; interaction *F*(2,37) = 11.42, *p* < 0.001).

### 3.4. DHA (PhA:50) Decreases Tau Hyperphosphorylation in the Hippocampus of ApoE^−/−^ Mice

Total tau protein expression was similar in all experimental groups (Figure 4A). Western blot analysis showed that levels of tau protein phosphorylated at serine 396 (p-tau^S-396^) were significantly higher in ApoE^−/−^ mice versus Control group (*p* < 0.001). In ApoE^−/−^ + DHA (PhA:50) and ApoE^−/−^ + DHA (PhA:1000) groups, the expression of p-tau^S-396^ decreased to control group values. No significant differences were found in p-tau^S-396^ protein expression between DHA (PhA:50) and DHA (PhA:1000) groups (Figure 4B). Figure 4B (two-way ANOVA: genotype *F*(1,38) = 3.39, NS; DHA *F*(2,38) = 11.84, *p* < 0.001; interaction *F*(2,38) = 25.55, *p* < 0.001).

CaMKII protein expression was significantly higher in ApoE^−/−^ group compared with Control mice (*p* < 0.05). ApoE^−/−^ + DHA (PhA:50) group showed decreased expression of CaMKII in hippocampus of ApoE^−/−^ mice versus ApoE^−/−^ group (*p* < 0.001). The hippocampal CaMKII level was normalized in ApoE^−/−^ + DHA (PhA:1000) with Control group (*p* < 0.001), but it was still significantly higher in ApoE^−/−^ + DHA (PhA:1000) group, than in the ApoE^−/−^ + DHA (PhA:50) group (*p* < 0.05). No significant differences were found in CaMKII expression in DHA (PhA:50) and DHA (PhA:1000) groups versus Control group (Figure 4C). Figure 4C (two-way ANOVA: genotype *F*(1,42) = 0.72, NS; DHA *F*(2,42) = 24.49, *p* < 0.001; interaction *F*(2,42) = 3.98, NS).

### 3.5. DHA (PhA:50) Increases Hippocampal BDNF Protein Expression Compared to DHA (PhA:1000) in ApoE^−/−^ Mice

Protein expression of BDNF was reduced (*p* < 0.01) in hippocampus of ApoE^−/−^ group compared with Control group. DHA (PhA:50) was able to increase (*p* < 0.05) reduced protein expression of BDNF in ApoE^−/−^ mice. But, ApoE^−/−^ + DHA (PhA:1000) group showed similar values of BDNF than ApoE^−/−^ group. No significant differences were found in BDNF expression in DHA (PhA:50) and DHA (PhA:1000) groups versus Control group (Figure 5). Figure 5 (two-way ANOVA: genotype *F*(1,39) = 29.48, *p* < 0.001; DHA *F*(2,39) = 9.49, *p* < 0.001; interaction *F*(4,39) = 5.54, *p* < 0.05).

### 3.6. DHA (PhA:50) Exerts Anti-Inflammatory Effect on Hippocampal ApoE^−/−^ Mice Compared to DHA (PhA:1000)

Protein expression of IL-6 was increased in hippocampus of ApoE^−/−^ mice compared with Control group (*p* < 0.01). DHA (PhA:50) reduced IL-6 hippocampal protein expression compared with ApoE^−/−^ mice (*p* < 0.01). Whereas, ApoE^−/−^ + DHA (PhA:1000) group showed enhanced (*p* < 0.05) levels of IL-6 compared to ApoE^−/−^ + DHA (PhA:50) group. Docosahexaenoic acid (PhA:50) and DHA (PhA:1000) groups showed decreased (*p* < 0.01) levels of this pro-inflammatory cytokine compared with the control group (Figure 6A). Figure 6A (two-way ANOVA: genotype *F*(1,42) = 82.28, *p* < 0.001; DHA *F*(2,42) = 72.20, *p* < 0.001; interaction *F*(2,42) = 11.30, *p* < 0.001).

ApoE^−/−^ mice showed increased hippocampal TNF-α protein expression compared with the control group (*p* < 0.01). Docosahexaenoic acid (PhA:50) was able to reduce increased protein expression of TNF-α in ApoE^−/−^ mice (*p* < 0.01). But, the ApoE^−/−^ + DHA (PhA:1000) group showed similar values of TNF-α than the ApoE^−/−^ group. No significant differences were found of TNF-α expression in DHA (PhA:50) and DHA (PhA:1000) groups versus the control group (Figure 6B). Figure 6B (two-way ANOVA: genotype *F*(1,42) = 1.17, NS; DHA *F*(2,42) = 13.20, *p* < 0.001; interaction *F*(2,42) = 9.69, *p* < 0.001).

High levels of iNOS protein expression were detected in the hippocampus of ApoE^−/−^ mice compared to the control group (*p* < 0.05). Protein expression of iNOS was lower in the hippocampus of ApoE^−/−^ + DHA (PhA:50) and ApoE^−/−^ + DHA (PhA:1000) mice compared to ApoE^−/−^ group (*p* < 0.01). No significant differences were observed in iNOS expression in the hippocampus of DHA (PhA:50) and DHA (PhA:1000) groups versus the control group (Figure 6C). Figure 6C (two-way ANOVA: genotype *F*(1,42) = 5.54, *p* < 0.05; DHA *F*(2,42) = 18.79, *p* < 0.001; interaction *F*(2,42) = 6.15, *p* < 0.005).

### 3.7. DHA (PhA:50) Decreases Hippocampal p22phox Expression and Antioxidant Response Compared to DHA (PhA:1000) in ApoE^−/−^ Mice

Oxidative stress induced by ApoE^−/−^ was reflected in increased p22phox protein expression when compared to the control. Docosahexaenoic acid (PhA:50) and DHA (PhA:1000) were able to reduce protein expression of p22phox, though low PhA-concentrated DHA treatment showed a better response to oxidative stress induced by ApoE^−/−^ than standard DHA did (Figure 7). Figure 7 (two-way ANOVA: genotype *F*(1,42) = 16.29, *p* < 0.001; DHA *F*(2,42) = 49.12, *p* < 0.0001; interaction *F*(2,42) = 22.23, *p* < 0.0001).

The ApoE^−/−^ group showed similar values of SOD-1 protein expression than the control group and both treatments, DHA (PhA:50) and DHA (PhA:1000), were able to reduce protein expression of SOD-1 in ApoE^−/−^ mice (*p* < 0.05). Similarly, no significant differences were observed in SOD-1 expression between DHA (PhA:50) and DHA (PhA:1000) groups versus the control group (Figure 8A). Figure 8A (two-way ANOVA: genotype *F*(1,33) = 1.02, NS; DHA *F*(2,33) = 46.93, *p* < 0.001; interaction *F*(2,33) = 2.86, NS).

The consistent results obtained by Western blot analysis showed the significantly increased expression of hippocampal catalase in the ApoE^−/−^ group compared with the control group. This effect was reversed by DHA (PhA:50) and DHA (PhA:1000) treatments, although the hippocampal catalase level was still significantly higher in the ApoE^−/−^ + DHA (PhA:1000) group than in the ApoE^−/−^ + DHA (PhA:50) group (*p* < 0.001). No significant differences were found in catalase protein expression in DHA (PhA:50) and DHA (PhA:1000) groups versus the control group (Figure 8B). Figure 8B (two-way ANOVA: genotype *F*(1,42) = 34.16, *p* < 0.001; DHA *F*(2,42) = 78.66, *p* < 0.001; interaction *F*(2,42) = 2.53, NS).

Glutathione peroxidase (GPx) protein expression levels were similar in all experimental groups, but DHA (PhA:50) showed a tendency to reduced values of GPx in wild-type and ApoE^−/−^ mice (Figure 8C). Figure 8C (two-way ANOVA: genotype *F*(1,42) = 14.26, *p* < 0.001; DHA *F*(2,42) = 36.22, *p* < 0.0001; interaction *F*(2,42) = 7.80, *p* < 0.01).

### 3.8. DHA (PhA:50) Exerts Anti-Apoptotic Effect on Hippocampal ApoE^−/−^ Mice Compared to DHA (PhA:1000)

Compared to the control group, the hippocampal caspase-3 level increased significantly in the ApoE^−/−^ group. Whereas, the hippocampal protein expression of caspase-3 was markedly reduced in the ApoE^−/−^ + DHA (PhA:50) group compared with the ApoE^−/−^ group (*p* < 0.05). The ApoE^−/−^ + DHA (PhA:1000) mice showed a tendency to reduced values of caspase-3, but still significantly higher in the ApoE^−/−^ + DHA (PhA:50) group than in the ApoE^−/−^ + DHA (PhA:1000) group (*p* < 0.05). 

Protein expression of caspase-3 was significantly decreased in the DHA (PhA:50) group (*p* <  0.01), but there were no differences in the DHA (PhA:1000) group compared to the control group (Figure 9). Figure 9 (two-way ANOVA: genotype *F*(1,42) = 33.12, *p* < 0.0001; DHA *F*(2,42) = 39.03, *p* < 0.0001; interaction *F*(2,42) = 2.19, NS).

## 4. Discussion

The study provides data supporting the neuroprotective effect of low PhA-concentrated DHA in an AD experimental model, compared to a standard PhA-concentrated DHA treatment. 

In the present study, we found that the spontaneous locomotor activity in the ApoE^−/−^ mice was significantly decreased versus control groups. The finding agrees with previous reports where a reduction in the locomotor activity in the ApoE^−/−^ mice was found, using either a wheel-running or a circular hole board apparatus [51,52]. However, we did not observe a substantial prevention by DHA in the ApoE^−/−^ reduced locomotor activity. Actually, there are some controversies regarding the effectiveness of DHA to regulate the animal’s locomotor activity. For instance, some studies have shown an improvement in motor activity after DHA treatment in a stroke experimental model [53], whereas DHA was not effective in reverting the locomotor deficits induced by a model of Parkinson’s disease [54]. 

Regarding learning, although not significant statistical differences were reached, we found a slight worsening in the learning curve of the ApoE^−/−^ mice. The treatment with DHA (PhA:50) resulted in a significant reduction in the learning curve in the ApoE^−/−^ mice compared with the control group. The same effect was observed in the memory performance, again in the ApoE^−/−^ mice. The DHA (PhA:50) group exhibited a significant reduction in the time latency to find the hidden location of the escape platform in the Morris Water Maze test, and an increase in time spent on the annulus close to such platform. Both parameters reveal that the deficits caused by the ApoE^−/−^ genotype are prevented by DHA (PhA:50), but not by DHA (PhA:1000) treatment. It can be ruled out that neither visual or sensorial impairment nor differences in swimming or motivation are mediating these results, since no differences were observed between genotypes or DHA treatment in the latency to find the marked platform. However, only the DHA (PhA:50) treatment was effective within the ApoE^−/−^ mice but not in the control animals. The result suggests a role for DHA (PhA:50) as treatment in cognitive deficit pathologies rather than in memory improvement therapies [30,31,55]. Our findings are supported by a growing body of evidence which shows that memory is rather improved by DHA when the subjects suffer a severe neurological disease, such as AD [27,28], or in adults with mild cognitive impairment or age-related cognitive impairment [33]. One of the main contributions of this work is the demonstration that a low concentration of PhA may dramatically regulate the effects of DHA on cognition. It could be proposed, that some of the discrepancies found in the literature regarding the effects of DHA in memory should be revised taking into account the content of PhA provided with the DHA treatment.

Our results showed a great increase of systemic cholesterol levels in the ApoE^−/−^ genotype fed on a high-fat diet, which was significantly reduced by DHA (PhA:50) treatment, to a higher extent, than DHA (PhA:1000). One of the main characteristics of the ApoE knockout mice is the highly elevated level of cholesterol, which is directly related to cognitive dysfunction [56]. Besides, we observed decreased BDNF levels in ApoE^−/−^ animals which were restored with DHA (PhA:50) treatment, but standard PhA-concentrated DHA treatment was not able to induce such beneficial effects in our AD model. The results are in line with previous studies, as it has been observed that a high-fat diet decreases neurogenesis and synaptic plasticity in ApoE^−/−^ animals due to decreased levels of BDNF in the hippocampus of animals [57,58,59] which correlates with deficits in learning and memory [60,61]. Docosahexaenoic acid has been widely reported to increase BDNF expression in different brain damage experimental models [62,63,64,65]. But despite of the positive effects of DHA, in our work, we are showing that PhA might be impeding improved protein expression induced by the LCPUFA, and thus, blocking its neuroprotective effect. 

Furthermore, ApoE^−/−^ mice showed increased levels of pro-inflammatory mediators TNF-α, IL-6, and iNOS. Both treatments reduced iNOS protein expression, but in the case of TNF-α and IL-6, only ApoE+DHA (PhA:50) could reduce the inflammatory effect of ApoE^−/−^. Docosahexaenoic acid is known as a potent anti-inflammatory LCPUFA [66]. Additionally, it has been found that PhA enhances the generation of reactive oxygen species in brain cells [67]. It is recognized that the activation of microglia and resulting elevated levels of neurotoxic and pro-inflammatory mediators is associated with neurodegenerative disease, including AD and acute cerebrovascular stroke, characterized by increased oxidative stress and neuroinflammation [24,68]. Thus, decreased PhA presence in DHA treatments seems to be highly interesting in terms of reducing deleterious effects such as inflammation and oxidative stress induced by PhA.

Additionally, ApoE^−/−^ mice exhibited increased Aβ, APP, and tau hyperphosphorylation. The results show that NADPH oxidase subunit p22phox also increased in ApoE^−/−^ mice, and even though both treatments decreased its protein expression, DHA (PhA:50) induced a greater reduction when compared to standard DHA treatment. In addition, PhA is partly degraded by the peroxisomal β-oxidation [69]. Enzymatic defects of the peroxisomal β-oxidation or the uptake of fatty acids into peroxisomes results in enhanced serum and tissue levels of several fatty acids. Moreover, peroxisomal β-oxidation of fatty acids is also a source of the generation of reactive oxygen species (ROS), in particular H_2_O_2_ [70]. Pha interferes with electron transport as well. As a consequence of slowing down the impaired electron transport, mitochondrial ROS generation becomes increased.

It has been demonstrated that abnormal accumulation of Aβ can promote the formation of reactive oxidative species involving the activation of N-methyl-D-aspartate receptors [11]. Docosahexaenoic acid reduces Aβ production via multiple pleiotropic mechanisms, and thus, is highly important for correct balance between amyloidogenic and non-amyloidogenic APP processing [71]. As previously introduced, lipids are important regulators for lateral movement of proteins within the phospholipid bilayer, and therefore, critical for substrate/enzyme interaction [72]. Docosahexaenoic acid-containing phospholipids also incorporate in cellular membranes and have been shown to change the organization of sphingolipid/cholesterol lipid-raft membrane domains [71]. Therefore, disturbing or changing the lipid composition of the membrane might therefore play a crucial role in the pathogenesis and treatment of AD. It has been reported that PhA is able to disturb the integrity of neural cells [73], thus the lack of efficiency in DHA treatment of AD might be attributed to the presence of PhA which could be changing membrane lipid composition.

Another main deleterious effect of PhA is the impairing of Ca^2+^ homeostasis which highly disturb the integrity of neural cells [74]. It has been demonstrated that a cellular overload of PhA in hippocampal astrocytes, neurons, and oligodendrocytes leads to a complex array of toxic activities, including mitochondrial dysfunction and Ca^2+^ deregulation via involvement of the intracellular InsP3–Ca^2+^ signaling pathway [67]. The observed contribution of an intracellular Ca^2+^ signaling pathway suggests that the activation of a membrane receptor coupled to intracellular Ca^2+^ release by PHA might be involved. A probable receptor candidate is the free fatty acid receptor GPR40 (also known as FFAR1) which activated by Pha might be a proposed mechanism mediating deleterious DHA (PhA:1000) effects. It has been proposed that Aβ may promote cellular Ca^2+^ overload by inducing membrane-associated ROS and forming pores in the membrane [75,76]. There is noteworthy evidence that intracellular Ca^2+^ homeostasis is disrupted in AD and can exacerbate Aβ formation and promote tau hyperphosphorylation. Even though experimental studies have shown that DHA treatment is able to reduce CAMKII overexpression [77], presence of PhA might be abolishing DHA effect in CAMKII regulation. This is consistent with our results since treatment with DHA (PhA:50) reduced increased CAMKII protein expression in ApoE^−/−^ mice and was significantly different when compared to standard DHA treatment. Aberrant hyperphosphorylation of tau weakens its interaction with microtubules leading to destabilization of the structure of microtubule as well, which therefore facilitates the formation of tau tangles and damages the neuronal homeostasis [78,79]. A recent study concluded that the ability of resveratrol, a polyphenolic non-flavonoid compound, to protect against tau hyperphosphorylation and to stimulate the dephosphorylation of tau protein, is related with the inhibition of GSK-3β and CaMKII and the activation of protein phosphatase 2 (PP2A) ([69,70]). Protein phosphatase 2 is one of the main phosphatases that causes tau dephosphorylation. Resveratrol is a stimulator of PP2A activity reducing tau phosphorylation [80]. It could be proposed that low PhA concentration in DHA mitigates tau hyperphosphorylation by reducing increased CAMKII protein expression in ApoE^−/−^ mice compared to standard DHA treatment.

Furthermore, it has been observed that increased cytosolic Ca^2+^ concentration and mitochondrial depolarization as well as opening of the mitochondrial permeability transition pore induced by PhA are key events in the induction of apoptotic and necrotic events [81,82,83]. This effect might be reflected in the augmented protein expression of the apoptotic mediator caspase-3 in our ApoE^−/−^ mice. The protective effect of DHA (PhA:50) treatment and the negative effect of DHA (PhA:1000) was also observed again in the results. Docosahexaenoic acid has been reported to protect against caspase activation, a hallmark of apoptotic cell death. For instance, in an ischemic injured brain rat model, DHA administration was able to decrease capasae-3 activity [84]. In our results, the presence of PhA blunted the anti-apoptotic effect of DHA (PhA:50) leading to increased protein expression of caspase-3 in the ApoE^−/−^ + DHA (PhA:1000) group. 

We also aimed to study the antioxidant response of both DHA compositions in ApoE^−/−^ mice. Low PhA-concentrated DHA decreased antioxidant response in ApoE^−/−^ mice when compared to the ApoE^−/−^ + DHA (PhA:1000) group. No significant differences were observed between ApoE^−/−^ mice and the control in SOD-1 and GPx protein expression which might suggest that in our model, antioxidant response is not mediated by these factors. Even though, both DHA treatments showed lower levels of SOD-1 and GPx when compared to ApoE^−/−^. On the other hand, ApoE^−/−^ showed a potent antioxidant response mediated by catalase which was significantly attenuated by DHA (PhA:50) but not by DHA (PhA:1000). Previous studies have shown the potent antioxidant capacity of DHA [85,86]. On the contrary, as previously described PhA has been demonstrated to induce oxidative stress under stress conditions associated with AD [87,88]. Thus, it could be proposed that due to high antioxidant capacity of low PhA-concentrated DHA, ApoE^−/−^ mice seems to be protected against oxidative processes. In this case, antioxidant response would not be such needed against the pro-oxidant effect of the ApoE^−/−^. On the contrary, increased production of oxidant agents by PhA together with the capacity to block DHA’s beneficial effect would be inducing a potent antioxidant response by the ApoE^−/−^ mice.

To summarize, low PhA-concentrated DHA decreased Aβ, APP, p-tau, BDNF, CAMKII, caspase 3, and catalase when compared to standard PhA-concentrated DHA. Furthermore, low PhA-concentrated DHA increased BDNF protein expression. Low PhA-concentrated DHA also protected against increased pro-inflammatory mediators IL-6 and TNF-α protein expression when compared to standard PhA-concentrated DHA. No significant differences were found between DHA treatments in p22phox, iNOS, GPx, SOD-1, and tau protein expression.

Concluding, in view of the results, it could be proposed that despite the positive effects of DHA treatments, the presence of PhA in DHA compositions might not only be reducing or blunting omega-3 effects but also stimulating deleterious effects itself. The positive actions of a low PhA-concentrated DHA were functionally reflected by improving the cognitive deficit in the AD experimental model. Therefore, since PhA is usually found in DHA compositions, reduction of PhA content would highlight a novel pathway for the neurodegeneration processes related to AD.

## Figures and Tables

**Table 1:** Effects of DHA (PhA:50) and DHA (PhA:1000) on serum concentrations of total cholesterol and low-density lipoprotein cholesterol (LDL c) in ApoE^−/−^ and wild-type mice.

(mg/dL)	Control	DHA (PhA:50)	DHA (PhA:1000)	ApoE^−/−^	ApoE^−/−^ + DHA (PhA:50)	ApoE^−/−^ + DHA (PhA:1000)
**Total cholesterol**	138.7 ± 22.27	85.33 ± 3.42 *	77.25 ± 5.42 *	570.4 ± 7.90 ***	473.4 ± 23.33 ***^###^	502.6 ± 15.74 ***^###^
**LDL-c**	54.40 ± 16.82	29.40 ± 11.24	36.77 ± 14.28	361.0 ± 62.35 ***	314.3 ± 48.40 ***^###^	304.9 ± 48.62 ***^###^

Data are presented as mean ± standard error of the mean (SEM). * *p* < 0.05 vs. control; *** *p* < 0.001 vs. control ^###^ *p* < 0.001 vs. ApoE.

**Table 2:** Effects of DHA (PhA:50) and DHA (PhA:1000) on body weight and increase body weightin ApoE^−/−^ and wild-type mice.

	Control	DHA (PhA:50)	DHA (PhA:1000)	ApoE^−/−^	ApoE^−/−^ + DHA (PhA:50)	ApoE^−/−^ + DHA (PhA:1000)
**Body weight (g)**	30.11 ± 0.92	29.58 ± 0.53	27.81 ± 0.52	30.48 ± 1.73	28.58 ± 0.45	29.32 ± 0.76
**Increased Body weight (g)**	10.15 ± 0.88	11.11 ± 0.50	8.71 ± 0.51	10.02 ± 0.76	8.45 ± 0.66	9.01 ± 0.96

Data were presented as mean ± standard error of the mean (SEM).

## Supplementary Materials

Supplementary Materials: The following are available online at http://www.mdpi.com/2072-6643/11/1/11/s1, Table S1: Diets composition.
