# Supplementary figures and images for: Low Phytanic Acid-Concentrated DHA Prevents Cognitive Deficit and Regulates Alzheimer Disease Mediators in an ApoE−/− Mice Experimental Model
Source: Nutrients. 2018 Dec 20;11(1):11. doi: 10.3390/nu11010011 (PMC6356727; doi:10.3390/nu11010011)

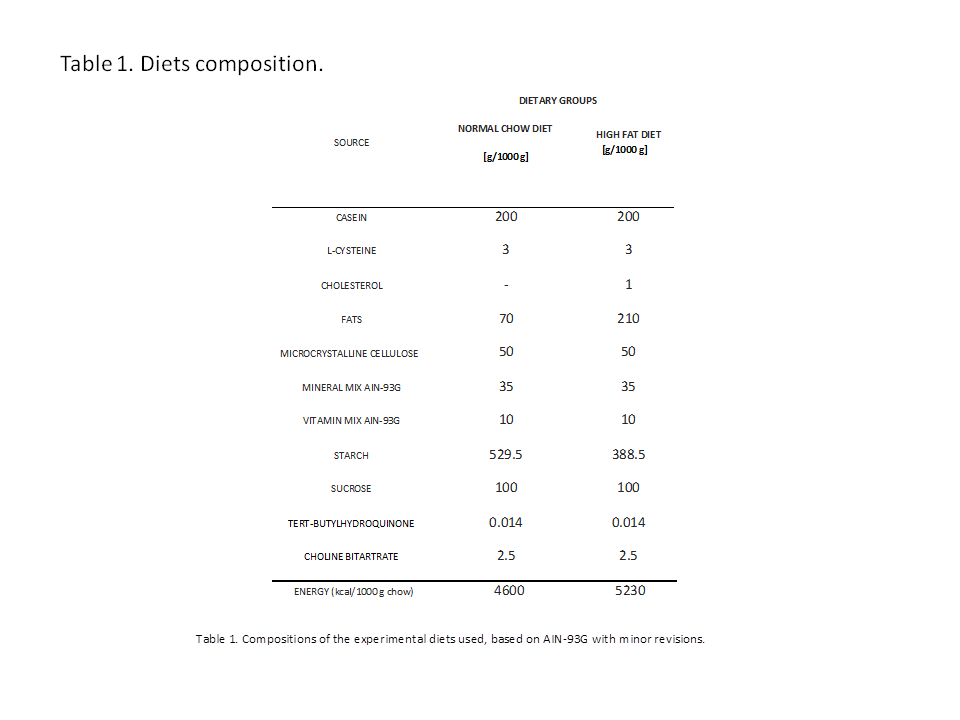

Supplement: Supplementary file 1 [file nutrients-11-00011-s001.zip › nutrients-402502-Supplementary Materials.tif]
